# Sources of information used by women during pregnancy and the perceived quality

**DOI:** 10.1186/s12884-022-04422-7

**Published:** 2022-02-08

**Authors:** Maaike Vogels-Broeke, Darie Daemers, Luc Budé, Raymond de Vries, Marianne Nieuwenhuijze

**Affiliations:** 1 Research Centre for Midwifery Practice, Zuyd University of Applied Sciences, Maastricht, Netherlands; 2 CAPHRI School for Public Health and Primary Care, Maastricht University, Maastricht, Netherlands

## Abstract

**Background:**

Access to reliable information is critical to women’s experience and wellbeing during pregnancy and childbirth. In our information-rich society, women are exposed to a wide range of information sources. The primary objective of this study was to explore women’s use of information sources during pregnancy and to examine the perceived usefulness and trustworthiness of these sources.

**Method:**

A quantitative cross-sectional study of Dutch women's experiences with various information sources during pregnancy, including professional (e.g. healthcare system), and informal sources, divided into conventional (e.g. family or peers) and digital sources (e.g. websites or apps). Exploratory backward stepwise multiple regression was performed to identify associations between the perceived quality of information sources and personal characteristics.

**Results:**

A total of 1922 pregnant women were included in this study. The most commonly used information sources were midwives (91.5%), family or friends (79.3%), websites (77.9%), and apps (61%). More than 80% of women found professional information sources trustworthy and useful, while digital sources were perceived as less trustworthy and useful. Personal factors explain only a small part of the variation in the perceived quality of information sources.

**Conclusion:**

Even though digital sources are perceived as *less* trustworthy and useful than professional and conventional sources, they are among the most commonly used sources of information for pregnant women. To meet the information needs of the contemporary generation of pregnant women it is essential that professionals help in the development of digital information sources.

## Background

Access to reliable information is critical to women’s experience and wellbeing during pregnancy and childbirth [1, 2]. Information and education help women understand what *is* happening and what *can* happen during this life-changing passage [3] and it improves women’s satisfaction with the childbirth experience [4]. Pregnant women seek information to feel more confident and comfortable in their communication with healthcare providers, to make decisions during the perinatal period, and to prepare themselves for their maternal responsibilities [4–8].

Adequate information helps to decrease stress and anxiety, provide support, and enhance self-esteem and internal control [9–12]. While inadequate information – either limited, contradictory, or false – is related to loss of control and limited participation in decision-making [5, 13, 14]. Not meeting women’s information needs during pregnancy can increase their worries and anxiety, is a risk factor for isolation, and is a predictor of low confidence as a parent [15]. Therefore, it is important for pregnant women to have access to information suited to their needs, delivered in the right amount and at the right time [9, 16, 17].

Fulfilling a woman’s information needs depends on her access to adequate resources and her ability to comprehend what has been presented to her [7]. In the current context of our information-rich society, women are exposed to a wide range of information sources. This includes information sources from the healthcare system, conventional sources (e.g. family, peers, and books) and digital information sources (e.g., websites, apps, and social media) [18, 19].

A woman’s use and appreciation of information depends on its quality, an assessment influenced by concepts of perceived trustworthiness and usefulness [20, 21]. While women express a desire for accurate information [19], they are aware that what they encounter may be inaccurate or biased. The trustworthiness of information is a major concern for them [7]. Two antecedents of trust in health information are defined1) “trust as the evaluation of information quality” or 2) “the intention to use the found information” [22] Because the possibly negative consequences of making decisions on untrustworthy or flawed information, trustworthiness of information is notably serious [23]

To assess trustworthiness of information, women look for information on one topic from a range of different information sources. If similar information is provided in different sources, they will perceive it as trustworthy [7, 19, 24]. A woman’s perception of the trustworthiness of information is associated with her health-beliefs, her age, and level of education [25]. However, even when information is perceived as trustworthy, it may not be considered useful [7]. Women judge the usefulness of information based on its appropriateness, evaluating it in the context of their personal circumstances, gestational age, personal beliefs, and values [7].

Although several studies have focused on women’s information seeking behaviors in maternity care, to our knowledge no study has compared formal, conventional and digital information sources, including women’s perceptions of their perceived trustworthiness and usefulness. Gaining more insight into the information sources pregnant women use to satisfy their information needs and how they perceive the quality of these sources will help healthcare providers to more effectively meet women’s preferences, contribute to improvement of decision-making based on correct information, and enhance the quality of woman-centred care. Therefore, the aim of this study is to describe women’s use of different sources of information and to examine how they perceive the quality of that information, based on their view of its usefulness and trustworthiness. We also explored the degree to which personal factors are associated with the perceived quality of different information sources.

## Methods

### Participants and settings

Data were obtained from StEM (Stem en Ervaringen van Moeders, [Voice and Experiences of Mothers]), a cross-sectional study of women's preferences and experiences during pregnancy, childbirth, and the postpartum period conducted in the Netherlands between February 2019 and February 2020.

Maternity care in the Netherlands is organised in primary and secondary levels of care. Community midwives offer primary care to healthy women with uncomplicated pregnancies, referring women to obstetrician-led hospital care when pathology is suspected or when complications occur. In obstetrician-led care, a woman may receive care from a hospital-based midwife, an obstetrician, or an obstetric resident, with an obstetrician having the final responsibility for care.

Women were invited to participate in the study through 81 midwifery practices and 7 hospitals across the Netherlands, and by social media. Women were eligible for this study if they were between 12 and 20 weeks pregnant (early pregnancy cohort), or if they were more than 32 weeks pregnant (late pregnancy cohort). Women could only participate once, either during early pregnancy OR late pregnancy.

Only women 18 years or older and with sufficient command of the Dutch language were included. We excluded women in cases of perinatal death or severe neonatal morbidity. Women gave their informed consent to participate and completed the questionnaire online, by post, or by telephone.

### Ethical considerations

The study was carried out in accordance with the Declaration of Helsinki. Women gave their informed consent to participate. The Human Research Ethics Committee of METC Z, Heerlen (registry number: METCZ20180121) approved the study.

### Measurement

We designed a self-administered questionnaire for each cohort. These questionnaires included validated tools, questions that had been used in previous studies, and additional questions about women’s background characteristics.

In this paper, we use data from the two cohorts described above. Women in each cohort were asked about their use of various information sources during pregnancy, including their perceptions of the trustworthiness and usefulness of those sources.

Women were asked to indicate which information sources they consulted during pregnancy. We distinguished professional sources from maternity care providers, so-called professional sources (midwives, obstetricians, general practitioner, leaflets from care providers, websites from midwifes/hospital, and information meetings organized by midwives/hospital), and informal sources divided into conventional sources (antenatal classes, family / friends, peers, books and journals) and digital sources (apps, websites about pregnancy and childbirth, forums and blogs, social media and TV and Netflix programs) (Table 1). Responses were measured on a 4-point Likert scale from never (1) to often (4). We then asked women to rate the perceived trustworthiness and usefulness of the sources. These were measured on a 5-point Likert scale from completely untrustworthy (1) to completely trustworthy (5), and completely useless (1) to completely useful (5).

**Table 1:** Categories in sources of information

**Professional sources**	
Midwives, obstetricians, general practitioner, leaflets from care providers, websites from midwifery practice or hospital, and information meetings organized by midwifery practice or hospital	
**Informal sources**	
*Conventional sources*	
Antenatal classes, family / friends, peers like other mothers and pregnant women, books and journals	
*Digital sources*	
Apps, websites about pregnancy and childbirth, forums and blogs, social media, and TV and Netflix programs	

We also collected data on psychological wellbeing, birth beliefs, social- and informational support, main healthcare provider, parity, age, level of education, marital status, and ethnicity.

The *Patient Health Questionnaire (PHQ-4)* was used to measure psychological wellbeing [26]. The PHQ-4 is a validated self-report questionnaire that consists of a depression scale (PHQ-2) and an anxiety scale (GAD-2). The composite PHQ-4 total score ranges from 0 to 12. Higher scores on the PHQ-4 represent higher levels of depression and/or anxiety.

The *Birth Beliefs Scale* was used to measure women's basic beliefs about birth as a natural or medical process [27]. This validated scale consists of two subscales: beliefs that birth is a natural process (five statements) and beliefs that birth is a medical process (six statements), rated on a 5–point Likert scale. Higher scores indicate stronger beliefs about birth as a natural or medical process.

The *Patient Reported Outcomes Measurement Information System (PROMIS*) was used to measure (1) informational support as perceived availability of helpful information or advice, and (2) social support as perceived feelings of being cared for and valued as a person [28]. Each concept of support was measured with four items scored on a 5-point Likert scale with higher scores indicating more support.

### Data analyses

Data are presented using frequencies and percentages for categorical variables and means and standard deviations (SDs) for continuous variables. Distributions of data about women’s uses and perceived trustworthiness and usefulness of information sources are reported using percentages.

We used backward stepwise multiple regression to analyse associations between personal characteristics and reported quality of information sources. The dependent variable was perceived quality, based on a summation (range 2-10) of the usefulness and trustworthiness of the source. The included predictor variables were age, psychological wellbeing, birth beliefs, social and informational support, stage of pregnancy (early or late), level of education (low, medium, high), and parity (nulliparous and multiparous). Categorical variables were recoded into dummy variables. Missing values were designated to system missing and excluded from analyses. *P*-Values of <0.05 were considered statistically significant. The data were analysed using IBM SPSS Statistics for Windows version 23.0.

## Results

Questionnaires were distributed to 2630 pregnant women (978 in early, and 1652 in late pregnancy). In total, 2091 women returned the questionnaire, 808 (82.6 %) in early and 1283 (77.7 %) in late pregnancy (total response rate 79.5%). In total, 169 questionnaires (58 in early, 111 in late pregnancy) were not complete. This resulted in 1922 questionnaires for analysis (750 in early, and 1172 in late pregnancy). The characteristics of pregnant women who participated are presented in Table 2.

**Table 2:** Characteristics of the participants

Characteristics	Early pregnancy (12-20 weeks) *n=750*		Late pregnancy (≥32 weeks) *n=1172*		Characteristics general Dutch population*
	n	(%)	n	(%)	
**Parity**^**1**^					
Nulliparous	258	(34.4)	441	(37.6)	43.9%
Multiparous	492	(65.6)	731	(62.4)	56.1%
**Age**^**1**^	Mean 30.4 years		Mean 30.4 years		N/A
	min 19 - max 43 years		min 18 – max 43 years		
< 20 years	2	(0.3)	5	(0.4)	0.7%
20-24 years	61	(8.1)	107	(9.1)	7.6%
25-29 years	254	(33.9)	393	(33.5)	29.3%
30-34 years	313	(41.7)	454	(38.7)	40.0%
35-39 years	106	(14.1)	194	(16.6)	18.8%
40-44 years	14	(1.9)	19	(1.6)	3.4%
**Level of education**^**2**^					
Low	46	(6.1)	58	(4.9)	9.9%
Middle	293	(39.1)	431	(36.8)	35.2%
High	410	(54.7)	683	(58.3)	53.7%
**Marital status**					
Married / living together	720	(96.0)	1141	(97.4)	N/A
Living apart together	6	(0.8)	5	(0.4)	N/A
Single	13	(1.7)	19	(1.1)	N/A
Unknown	11	(1.5)	7	(0.6)	N/A
**Nationality**					
Dutch	668	(89.1)	1037	(88.5)	N/A
Non-Dutch	82	(10.9)	134	(11.4)	N/A
Unknown			1	(0.1)	N/A
**Main healthcare provider**^**1**^					
Midwife	675	(90.0)	963	(82.2)	87.0% at start of antenatal care
Obstetrician	37	(4.9)	116	(9.9)	12.5% at start of antenatal care
Shared care	38	(5.1)	93	(7.9)	

*data source for characteristics of the general Dutch population.

^1^Peristat, Perinatale cijfers in Nederland, year 2019 [29]

^2^CBS Statline womens's level of education between 25-45 years [30]

### Information sources used during pregnancy

Almost all women in our study got information from a midwife at some point during pregnancy (early pregnancy 96.4% and late pregnancy 98.5%). Women were less likely to use other professional sources, like leaflets from care providers. Frequently used informal conventional information sources were peers, like pregnant women and other mothers (early pregnancy 86% and late pregnancy 91%), and family or friends (early pregnancy 92% and late pregnancy 93.3%).

**Fig. 1:**
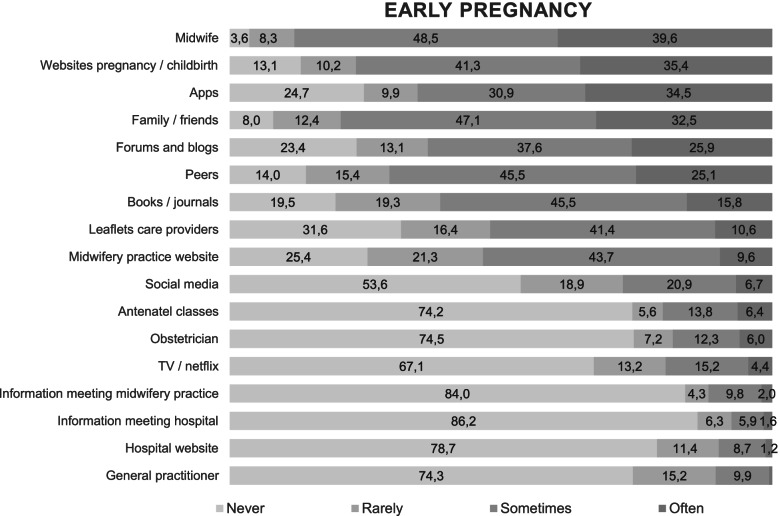
Information sources used during early pregnancy in percentage

**Fig. 2:**
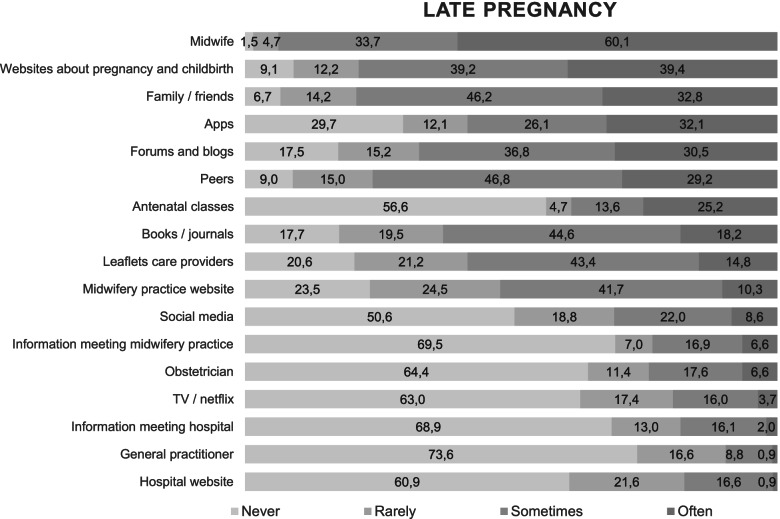
Information sources used during late pregnancy in percentage

A majority of women used digital sources, e.g. websites about pregnancy and childbirth (early pregnancy 86.9% and late pregnancy 90.9%) or apps (early pregnancy 75.3% and late pregnancy 70.3%), whereas social media - e.g. Twitter, Facebook, and Instagram - were less commonly used (Figs. 1 and 2).

### Perceived trustworthiness and usefulness

**Fig. 3:**
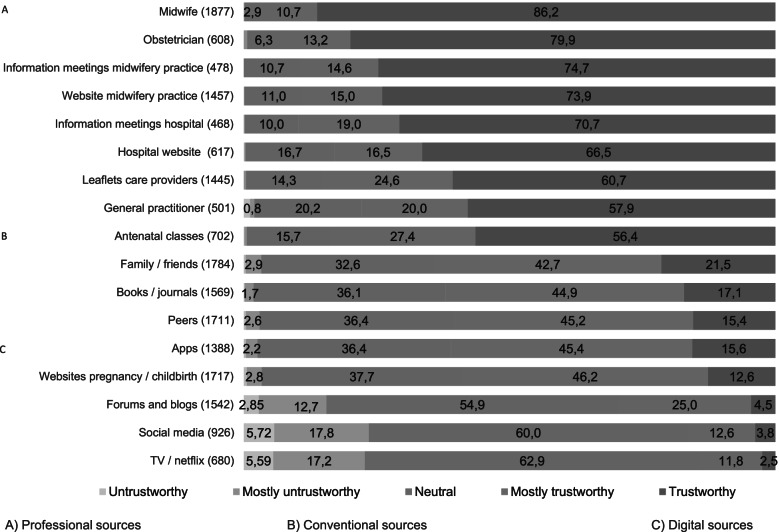
Trustworthiness of information sources in percentage

**Fig. 4:**
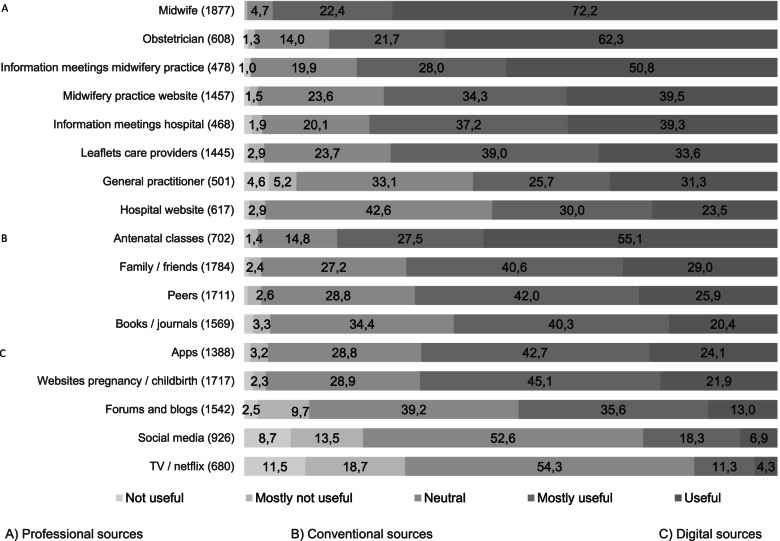
Usefulness of information sources in percentage

We asked women to rate the trustworthiness and usefulness of the sources they used, (Figs. 3 and 4). Women expressed a high level of trust in professional information sources. More than 90% of all women identified their care provider (midwife or obstetrician) as a trustworthy source of information, while conventional sources like peers were given lower scores of trustworthiness. Digital information was perceived as least trustworthy (Fig. 3).

Most of the professional and conventional sources scored higher than digital sources on usefulness. More than 80% of women found information from their midwife, obstetrician, and antenatal classes (completely) useful, while about 60% found apps and websites to be useful (Fig. 4).

### The effect of personal factors on use and perceived quality of information sources

We looked more closely at frequently used information sources in relation to women’s personal characteristics (Table 2). Additionally, we looked at the association between personal characteristics and the perceived quality of information sources (Table 3). We focused on one professional information source (leaflets from care providers), and four informal information sources, including two conventional sources (antenatal classes, and peers) and two digital sources (websites and apps), because all of these sources require active information seeking behaviour of women.

**Table 3:** Information sources and characteristics of frequent users

Characteristics N=total 1922	Leaflets care providers		Antenatal classes		Peers		Websites pregnancy and childbirth		Apps	
	N=1072*		N=605*		N=1419*		N= 1498*		N= 1172*	
	**N**	% **	**N**	% **	**N**	% **	**N**	% **	**N**	% **
**Phase of pregnancy**										
Early (750)	390	52.0%	151	20.1%	528	70.6%	576	76.8%	489	65.4%
Late (1172)	682	58.2%	454	38.7%	891	76.0%	922	78.7%	682	58.2%
**Main healthcare provider**										
Midwife (1638)	921	56.0%	523	31.9%	1212	74.0%	1278	78.0%	1007	61.5%
Obstetrician (153)	78	51.0%	40	26.1%	114	74.5%	122	79.7%	89	58.2%
Shared care (131)	73	55.7%	42	32.1%	93	70.0%	98	74.8%	76	58.0%
**Parity**										
Nulliparous (699)	452	64.7%	294	42.1%	584	83.5%	589	84.3%	493	70.5%
Multiparous (1233)	620	50.7%	311	25.4%	835	68.3%	909	74.3%	679	55.5%
**Age** *mean (SD)*	29.9	(4.31)	30.6	(4.08)	30.1	(4.22)	30.2	(4.36)	30.0	(4.15)
< 20 years (7)	5	71.4%	1	14.3%	6	85.7%	5	71.4%	4	57.1%
20-24 years (168)	116	69.0%	37	22.0%	126	75.0%	144	85.7%	107	63.7%
25-29 years (647)	384	59.4%	215	33.2%	508	78.5%	521	80.5%	440	68.0%
30-34 years (767)	420	54.8%	252	32.9%	557	72.6%	580	75.6%	453	59.1%
35-39 years (300)	127	42.3%	91	30.3%	204	68.0%	221	73.7%	154	51.3%
40-44 years (33)	20	60.6%	9	27.3%	18	54.4%	27	81.8%	14	42.4%
**Level of education**										
Low (104)	46	44.2%	20	19.2%	74	71.2%	71	68.3%	62	59.6%
Middle (724)	406	56.1%	172	23.8%	538	74.3%	562	77.6%	456	63.0%
High (1093)	619	56.6%	413	37.8%	807	73.8%	864	79.9%	653	59.7%
Unknown	1	0.1%					1	0.1%	1	0.1%
**Birth Belief Scale (Range 1-5)**										
Natural process										
Frequent users *mean (SD)*	3.83	(0.51)	3.96	(0.56)	3.83	(0.55)	3.81	(0.54)	3.80	0.55
Non-frequent users *mean (SD)*	3.81	(0.60)	3.76	(0.54)	3.82	(0.55)	3.89	(0.58)	3.86	(0.55)
Medical process										
Frequent users *mean (SD)*	3.03	(0.55)	2.92	(0.62)	3.04	(0.58)	3.06	(0.56)	3.06	(0.57)
Non-frequent users *mean (SD)*	3.04	(0.62)	3.09	(0.56)	3.00	(0.61)	2.94	(0.65)	2.99	(0.60
**PHQ (range 4-16)**										
Frequent users *mean (SD)*	5.45	(1.84)	5.33	(1.65)	5.45	(1.82)	5.50	(1.9)	5.52	(1.91)
Non-frequent users *mean (SD)*	5.42	(1.92)	5.49	(1.97)	5.41	(2.03)	5.22	(1.82)	5.30	(1.81)

* N is the sum of women who sometimes or often used a specific information source (=frequent user)

**The percentages express the proportion of women with that specific condition or characteristic who frequently used that source of information

Nulliparous women used all sources more frequently than multiparous women. The use of leaflets and websites was lower amongst women who had a low level of education versus a middle or high level of education. Antenatal classes were more often used by women with a high level of education compared to the other two levels. Women who used antenatal classes had higher mean scores on birth beliefs as a natural process and lower mean scores on birth beliefs as a medical process (Table 3).

The associations between personal factors and the perceived quality of information sources are presented in Table 4. Multiple linear regression showed that a limited number of personal factors were associated with the perceived quality of information sources.

**Table 4:** Association between personal factors and perceived quality of information sources

	Leaflets			Antenatal classes			Peers			Websites			Apps		
Predictors	*Unstandardized β*	Standardized Coefficients *β*	*p-*Value	*Unstandardized β*	Standardized Coefficients *β*	*p-*value	*Unstandardized β*	Standardized Coefficients *β*	*p-*value	*Unstandardized β*	Standardized Coefficients *β*	*p-*Value	*Unstandardized β*	Standardized Coefficients *β*	*p-*value
**Phase of pregnancy (late)**	0.166	0.056	0.033	0.637	0.192	0.000	-0.159	-0.054	0.024	**-**	**-**	**-**	**-**	**-**	**-**
**Parity (multiparous)**	**-**	**-**	**-**	**-**	**-**	**-**	-0.378	-0.129	0.000	-0.284	-0.099	0.000	-0.289	-0.097	0.001
**Age**	**-**	**-**	**-**	**-**	**-**	**-**	**-**	**-**	**-**	**-**	**-**	**-**	0.021	0.059	0.034
**Level of education**															
Low	-0.646	-0.090	0.001	**-**	**-**	**-**	**-**	**-**	**-**	**-**	**-**	**-**	**-**	**-**	**-**
Medium	-0.307	-0.105	0.000	**-**	**-**	**-**	**-**	**-**	**-**	**-**	**-**	**-**	**-**	**-**	**-**
**PHQ**	**-**	**-**	**-**	0.075	0.083	0.023	**-**	**-**	**-**	**-**	**-**	**-**	**-**	**-**	**-**
**Support**															
Social support	**-**	**-**	**-**	**-**	**-**	**-**	**-**	**-**	**-**	0.046	0.075	0.002	0.039	0.059	0.030
Informational support	0.047	0.086	0.001	**-**	**-**	**-**	0.087	0.158	0.000	**-**	**-**	**-**	**-**	**-**	**-**
**Birth Belief Scale**															
Natural	0.036	0.067	0.024	0.113	0.20	0,000	**-**	**-**	**-**	**-**	**-**	**-**	**-**	**-**	**-**
Medical	0.036	0.086	0.004	**-**	**-**	**-**	0.034	0.084	0.000	**-**	**-**	**-**	0.034	0.081	0.002
**Adjusted** ***R***^**2**^	3.0%		0.000	9.2%		0.000	5.3%		0.000	1.7%		0.000	1.7%		0.000

After stepwise backwards regression factors with **-** were not statistically significant predictors in the final model and therefore removed

The quality of the leaflets from maternity care professionals was rated higher by women in the late stage of pregnancy, with a high level of education, a higher level of informational support, and stronger birth beliefs (both natural and medical).

For antenatal classes, a lower level of psychological wellbeing (i.e. higher levels of anxiety and depression), a higher score on birth beliefs as a natural process and being in the late stage of pregnancy were significantly associated with higher perceived quality.

The quality of information from peers – such as pregnant women and other mothers – was rated higher by nulliparous women, women in early pregnancy, and women with higher levels of informational support.

The quality of websites was rated higher by nulliparous women, women with higher levels of social support, while apps were rated higher by nulliparous women who were older, with a higher level of social support and higher beliefs about birth as a medical process.

## Discussion

Our study investigated the information sources used by women during pregnancy including their perceptions of the quality of that information, as measured by its reported trustworthiness and usefulness.

We found that midwives were the most frequently used source of information, followed, in order, by informal sources such as websites, pregnancy and childbirth apps, family and friends, forums, blogs and peers. Social media (e.g., Twitter and Facebook) were less often used to gain information. The number of women using obstetricians as information source is much lower in our study. We need to keep in mind that, in our sample, 95% of the women in early pregnancy and 82% of the women in late pregnancy received care from a midwife, while only 10% and 18% respectively received care from an obstetrician. These percentages reflect the care given to the whole pregnant population in the Netherlands.

Our findings are consistent with the results of a systematic literature review of 31 studies from 14 countries that found the most common information sources used by pregnant women to be health professionals, family, friends, and the internet [31]. Despite growing interest in digital sources among pregnant women in the Netherlands, the midwife as professional source was the most widely used source of information for pregnant women [32].

Despite the high use of digital sources, such as websites and apps, women in our study rated these media as the least trustworthy sources of information. Professional sources were regarded as more trustworthy and seen as offering more useful information. Previous researchers have already suggested that it is unlikely that digital sources will replace the importance of the “human touch” of healthcare professionals [33]. As Camacho [32] points out, healthcare providers provide reassurance when pregnant women are confronted with contradictions in other information sources.

Other studies found that digital sources have a more complementary function, used by women as an extra source of information outside the healthcare system [34, 35]. Easy accessibility and unlimited availability of digital information makes it a convenient source of additional information [15, 24, 36, 37]. A study in the Netherlands reported that the minority of women who did not use the internet as an information source during pregnancy did not feel the need to do so as long as they received enough information from other sources [24]. There is some concern that women who use the internet as an information source for decisions concerning pregnancy and childbirth [24, 38, 39] rarely discuss that information with their maternity care providers [40, 41]. Since our study pointed out a high use of digital sources, midwives should ask women what information sources they are using for their decision-making and be prepared to recommend websites that are trustworthy and useful. By initiating conversations about the reliability of information sources, care providers can prevent inaccurate decisions based on misinformation, while, at the same time, strengthening the process of shared decision-making.

Social media may be regarded as less trustworthy because they are designed for social networking and support [19]. Social media create communication platforms where women may connect with other pregnant women to share experiences and acquire emotional or informational support [37, 42]. Still, over time these media may become more influential as women appreciate information from interpersonal sources, especially from people like themselves [43].

Compared to women with middle and high levels of education, women with a low level of education use written information sources like leaflets and websites less often. Higher levels of health literacy are often essential to obtain, understand, assess, and use health-related information and to make health-related decisions [44]. People with lower levels of health literacy are more likely to prefer text-limited sources to receive health information [45]. Using visual images next to plain language can lead to a better understanding of health information during pregnancy [7]. Even if there is equal access to leaflets and websites, the use of complicated language will limit its value to women with limited health literacy.

Another important finding of our study is that leaflets provided by maternity care professionals are used less often than peers, apps, and websites. An earlier Dutch study reported that women are given too many leaflets and they do not address the information needs of women in a “just-in-time” manner [9]. This may explain what we learned about the limited use of leaflets, regardless the educational level of the women.

We found that nulliparous women used a larger variety of information sources during their pregnancy than multiparous women. Most likely nulliparous women have higher information needs, because of the novelty of this life changing period. Our results are in line with the results of a previous study of Kamali [6] who reported that being a nulliparous women had a significant effect on the use of information sources, while multiparous women relied more on their prior knowledge and experience.

Personal factors explain only a small part of the variation in perceived quality of information sources, especially digital information sources. Personal factors account for only 1.7% of the variation in both the perceived quality of websites and the perceived quality of apps. We know from other studies that people judge the usefulness and trustworthiness of health information sources based on several features of that information including: 1) the authority or professional source of information; 2) regency of the information; 3) use of plain language; 4) details of information; 5) customised or personalised information; 6) reassurance; 7) lack of bias; 8) inclusion of further contacts and sources for help 9) attractive and colourful design, and 10) user-friendliness, e.g. easy and immediately accessible [20, 21, 37]. However, such in-depth investigation about the features of the information sources was beyond the scope of this study. Further research on the drivers of perceived quality of information sources should use multiple items to measure characteristics of the information and of the users.

### Study strengths and limitations

Our study has both strengths and limitations. To our knowledge, this is the first study that explores both the usefulness and trustworthiness of information sources used by Dutch pregnant women, including professional sources and informal sources like digital sources and conventional sources. Furthermore, our results are based on a large sample of 1922 women spread throughout the Netherlands. Our study is limited by the fact that we had little direct control over the inclusion process. We do not know the exact number of women eligible for this study, and we do not have information about non-responders and women who refused to participate. Because part of our participants were invited through social media (like Facebook and Twitter), it may be that our study population uses digital media more frequently than the general population of Dutch pregnant women. However, a vast majority of our participants (90.4%) were recruited by healthcare providers and not via the internet. Like many survey studies, our participants are not completely comparable with the general Dutch population of pregnant women. The level of education of participants was slightly higher and we had more multiparous than nulliparous women in our study. Furthermore, the questionnaires were only available in the Dutch language, resulting in under-representation of ethnic minorities. Finally, it was beyond the focus of our study to explore men’s experiences, even though we know that the opportunity to receive information addressing the needs and perspectives of fathers supports the transition to fatherhood [46, 47].

## Conclusion

Professional sources of information, are perceived as highly trustful and useful. Interestingly, digital sources are one of the most commonly used information sources by pregnant women, even though they are perceived as less useful and trustworthy than professional sources. Midwives, as the most common main providers of maternity care in the Netherlands, are highly valued as an important personal source of information. We also found that the perceived quality of different sources of information did not vary across different characteristics of our participants, suggesting that many additional factors play a role in the assessment of the quality of information. Our research points to the need to put more emphasis on developing professional information about pregnancy and childbirth in digital sources like websites and apps, as it seems that leaflets do not match the information needs of the contemporary generation of pregnant women. In their contacts with pregnant women,

pregnancy and childbirth and guide women to trustworthy and useful digital information sources. Through these discussions maternity care providers can prevent inaccurate decisions based on misinformation, while strengthening the process of shared decision-making.

## Data Availability

The datasets used and/or analysed during the current study are available from the corresponding author on reasonable request.
